# Discovery and characterization of novel DNA viruses in *Apis mellifera*: expanding the honey bee virome through metagenomic analysis

**DOI:** 10.1128/msystems.00088-24

**Published:** 2024-03-05

**Authors:** Dominika Kadlečková, Martina Saláková, Tomáš Erban, Ruth Tachezy

**Affiliations:** 1 Department of Genetics and Microbiology, Faculty of Science BIOCEV, Charles University, Vestec, Průmyslová, Czechia; 2 Crop Research Institute, Drnovská, Prague, Czechia; Dalhousie University, Halifax, Canada

**Keywords:** metagenomics, honey bee viruses, virome, virus discovery, honey bee

## Abstract

To date, many viruses have been discovered to infect honey bees. In this study, we used high-throughput sequencing to expand the known virome of the honey bee, *Apis mellifera*, by identifying several novel DNA viruses. While the majority of previously identified bee viruses are RNA, our study reveals nine new genomes from the *Parvoviridae* family, tentatively named Bee densoviruses 1 to 9. In addition, we characterized a large DNA virus, Apis mellifera filamentous-like virus (AmFLV), which shares limited protein identities with the known Apis mellifera filamentous virus. The complete sequence of AmFLV, obtained by a combination of laboratory techniques and bioinformatics, spans 152,678 bp. Linear dsDNA genome encodes for 112 proteins, of which 49 are annotated. Another large virus we discovered is Apis mellifera nudivirus, which belongs to a group of *Alphanudivirus*. The virus has a length of 129,467 bp and a circular dsDNA genome, and has 106 protein encoding genes. The virus contains most of the core genes of the family *Nudiviridae*. This research demonstrates the effectiveness of viral binning in identifying viruses in honey bee virology, showcasing its initial application in this field.

**IMPORTANCE:**

Honey bees contribute significantly to food security by providing pollination services. Understanding the virome of honey bees is crucial for the health and conservation of bee populations and also for the stability of the ecosystems and economies for which they are indispensable. This study unveils previously unknown DNA viruses in the honey bee virome, expanding our knowledge of potential threats to bee health. The use of the viral binning approach we employed in this study offers a promising method to uncovering and understanding the vast viral diversity in these essential pollinators.

## 
INTRODUCTION

The honey bee (*Apis mellifera* Linnaeus, 1758) is an indispensable global pollinator, playing a pivotal role in global agriculture and ecosystem dynamics (1, 2) and is a model organism for biological research on eusocial insects (3, 4). Therefore, it is alarming that the high annual losses of honey bee colonies continue around the world. The overwintering losses in some countries exceed 30%, but in some regions, losses exceeding 50% have been reported (5, 6). Many different factors have been described that could account for these colony losses and most likely a combination of several of them is the cause (6, 7). One of the many factors is viral infections (8), which may interact with diverse biotic and abiotic stressors (9). Although the focus is often on pesticides, the main threat is *Varroa destructor* which triggers viral infections in honey bees (10, 11). It is important to consider that viruses are a common and abundant part of the bee colony, similar to the more thoroughly studied bacterial microbiome.

All viruses found in certain spaces are called viromes. In bees, the virome consists of bee-infecting viruses, viruses infecting other eukaryotes living in/on bees (viruses of parasites), bacteriophages, and transient viruses present in pollinator resources such as pollen (12). Several new bee viruses have been discovered using next-generation sequencing (NGS) techniques (13–15). Thanks to NGS, approximately 72 viruses have been described in honey bees (16). Most of viruses found in honey bees belong to the +ssRNA group, such as the *Iflaviridae* and *Dicistroviridae*. So far, only a few DNA viruses have been identified: the large dsDNA virus Apis mellifera filamentous virus (AmFV), discovered in 1978 (17) but fully sequenced in 2015 (17, 18), and the recently discovered small single-stranded DNA viruses belonging to the families *Genomoviridae* and *Microviridae* (19).

In our recent project focusing on the honey bee virome, we found sequences of bee DNA viruses belonging to the *Parvoviridae* family, a large DNA virus related to the AmFV virus, and another to *Nudiviridae*. Parvoviruses, especially those belonging to the subfamily *Densovirinae*, are well-known small nonenveloped viruses that infect insects (20). The genome of parvoviruses consists of linear ssDNA, 4–6 kb long, which contains two major expression cassettes with open reading frames (ORFs) that code non-structural proteins and structural capsid proteins (21, 22). The known members of this family are highly pathogenic for their insect hosts (20). The other sequences we have identified are similar to a large dsDNA AmFV with a genome of over 450 kb. This virus has not yet been classified and some of its proteins show identities with proteins of the family *Baculoviridae* (18). Individuals with high viremia have milky hemolymph due to cellular degradation caused by the presence of virions and show signs of weakness in crawling bees at the entrance. Although the virus is widespread in colonies in different parts of the world (23, 24), clinical symptoms are detected only sporadically (17, 25). *Nudiviridae* are also a group of viruses that infect insects and crustaceans; they are enveloped dsDNA viruses with a large genome of 90–230 kb (26).

In this study, we, for the first time, describe bee parvoviruses, Apis mellifera filamentous-like virus and Apis mellifera nudivirus (AmNV). Laboratory and bioinformatic approaches were combined to complete the genomes of these new honey bee viruses. Viral binning and the creation of vMAGs (viral metagenome-assembled genomes) were used for the first time, to our knowledge, for the genome assembly of honey bee viruses.

## MATERIALS AND METHODS

### *De novo* sequencing

#### Honey bee collection, sample processing, and libraries preparation

Bees were collected from 18 hives in five different locations in Czechia [Lisnice, Libechov, Prasily, Brdy/Nerezin, and Prague-Ruzyne in the Crop Research Institute (abbreviated as VURV)]. In addition, two of the five VURV colonies used for analyses were moved from a different site out of the flying range to a demarked place in VURV two weeks before sampling. Immediately after sampling by shaking from a brood comb frame into a plastic bag, they were placed in a polystyrene box on dry ice. Then, the bees were divided to sterile centrifugal tubes and stored at −80°C until further use. Except for two colonies, which had obvious signs of varroosis, i.e. crippled wings, all hives were denoted to be “healthy,” that is, the colonies had rapid build-up, showed no damaged capping, and no signs of overt diseases or *Varroa* infestation were observed. The list and specifications of the samples are available on GitHub (https://github.com/kadlck/NAZV19).

**Fig 1:**
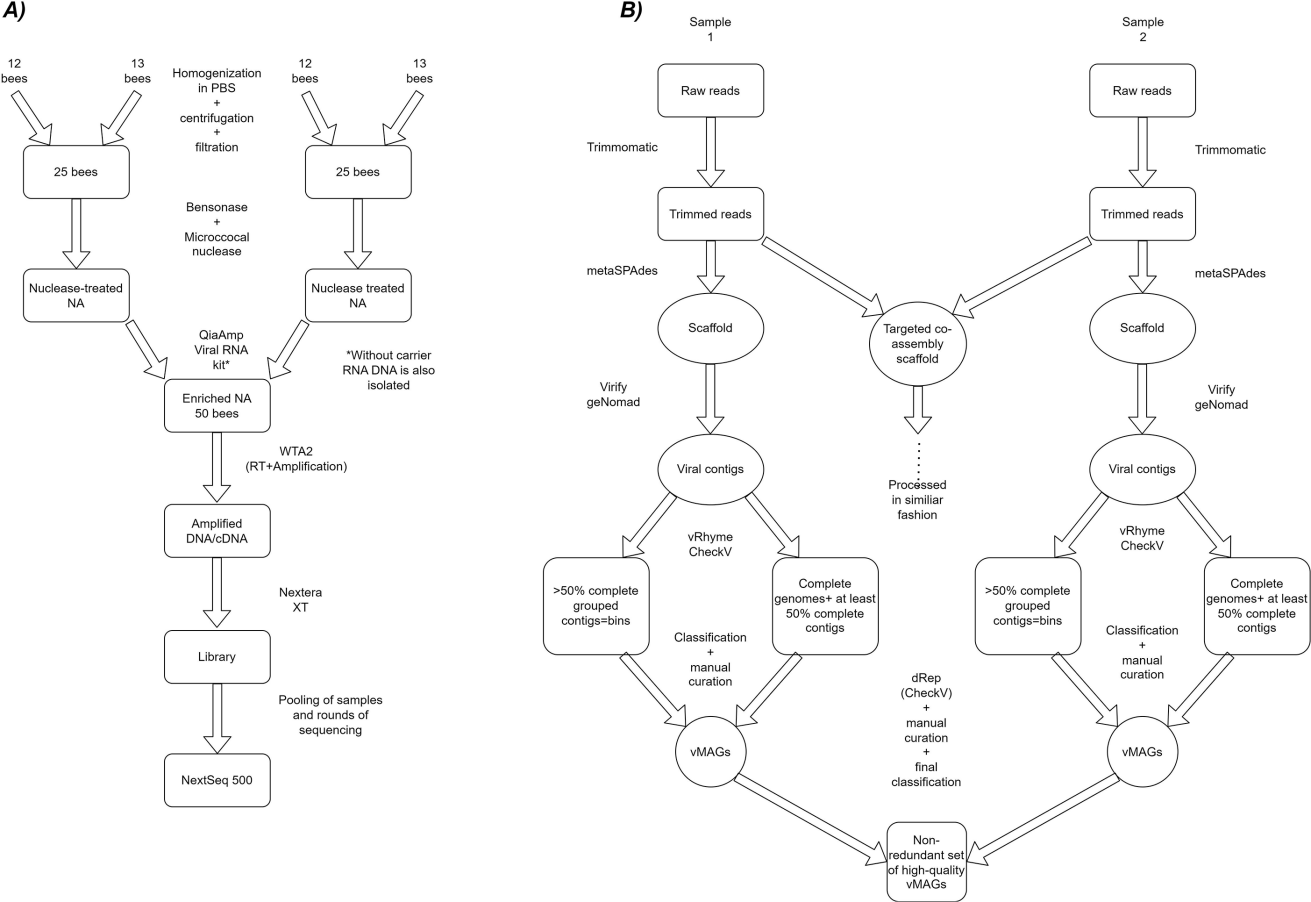
Overview of the workflow of sample preparation (A) and bioinformatical procedure (B) used in this study.

Fifty randomly selected bees from each hive were pooled in one sample. As we suggested in (24) this number of bees in the sample should allow for capturing the full diversity of eukaryotic viruses in the colonies. We processed the bees as described in detail before (24). Briefly, the homogenization was done in four 5mL tubes with ceramic beads (Bertin technologies, Montigny-le-bretonneux, Ile-de-France, France), after centrifugation and filtration the bees were pooled into two separate aliquots of 25 bee pools, and after extraction of encapsulated nucleic acids with QIAamp Viral RNA Kit (Qiagen, Hilden, Germany), the two 25 bee pools were combined back into one sample of 50 bees. The reverse transcription of RNA and amplification of cDNA/DNA was done with WTA2 kit (Sigma-Aldrich, St. Louis, Missouri, United States). Libraries were prepared with Nextera XT (Illumina, San Diego, California, USA). The sequencing was performed in several runs on NextSeq 500 using Mid Output Kits (Illumina, San Diego, California, USA) for 2 × 150 bp paired-end cycles, with minimum of 10M reads per sample. The 1xPBS was used as a negative control and has been processed through all the steps (from homogenization to sequencing) to exclude possible contamination of samples and reagents during processing (see Fig. 1 for overview).

#### Bioinformatics—data analysis

The quality of sequences was checked with FastQC v0.11.9 (https://www.bioinformatics.babraham.ac.uk/projects/fastqc). Trimming was performed with Trimmomatic v0.39.10 (27) using adapters/primers from both amplification steps with ILLUMINACLIP, HEADCROP:19, LEADING:15, TRAILING:15, SLIDINGWINDOW:4:20, and MINLEN:50. After trimming, the quality of the reads was checked again. The reads were assembled with SPAdes v3.15.3 (28) --meta using the following k-mers: k 21, 33, 55, 77, and the gained contigs were classified with Diamond v2.0.11 (29) blastx against the non-redundant (NR) database ((National Center for Biotechnology Information (NCBI) downloaded on 9 August 2021). The classification for each sample was displayed using Kronatools v2.8.1 (30). Contigs larger than 500 bp from all samples were clustered based on pairwise ANI (average nucleotide identity) with 95% identity over 85% length using scripts and instructions provided by CheckV v1.0.1 (31). Classification was done again using Diamond v2.0.11 (29) blastx, but with --sensitive and --c 1 settings. The reads were mapped back to the contigs using bwa-mem2 v2.2.1 (32) and the mapping of reads was extracted using CoverM v0.6.1 (https://github.com/wwood/CoverM).

We considered removing the host reads from the samples prior to analysis but they made up on average ~36% of all reads. When we compared the results of assembly of dehosted reads with those non-dehosted we found that the statistic of the scaffold was in very slight favour of non-dehosting option on most of the samples. Therefore, we continued with reads containing the host reads (Table 1 and full statistics on GitHub).

### Determination of the complete genome of Apis mellifera filamentous-like virus

#### Bioinformatics—the creation of MAGs

Since we found contigs belonging to large viruses, we tried binning and creating vMAGs, see Fig. 1. Several different steps and softwares were tried on test samples, and the best-performing software was used further. First, we predicted viral contigs from the scaffold (>1,500 bp) using Virify v0.4.0 (33) and geNomad v1.2.0 (https://github.com/apcamargo/genomad). Trimmed reads were mapped back to the viral contigs and then were binned using vRhyme v1.1.0 (34). The viral contigs were also checked with CheckV v1.0.1 (31), whose output was used for dRep v3.4.0 (35), removing bins with less than 50% completeness. The complete genomes (ITR/DTR/circular genomes) identified by vRhyme (34) and CheckV v1.0.1 (31) were extracted and added to the bins. Bins were checked for mismatched classifications [Diamond (29) against IMG/VR v4 (36) and NR, geNomad classification], and split if necessary. They were further split on the classification which we were able to designate with certainty, considering the percent of identity and CheckV statistics (contamination/warnings). Furthermore, the high-quality vMAGs/genomes from all samples were gathered, and CheckV v1.0.1 (31) with dRep v3.4.0 (35) was run again. This resulted in a non-redundant set of vMAGs that were at least 50% complete. They were classified against IMG/VR v4 (36) and the NR protein database (NCBI). All suspicious vMAGs (based on CheckV statistics and warnings; or with uncertain or disputable classification), with a focus on eukaryotic viruses, were checked again over the whole length of the sequence against the NR in BLAST at NCBI and manually curated. Most of these cases with difficult assignment/splitting were phages.

#### Completing vMAG with additional sequencing

For the processing of AmFLV), the sequences of the three largest contigs were extended by amplicon sequencing. Primers were designed at both ends of the three contigs (available on GitHub, see Data Availability for link), and PCR was performed with primer combinations using Phusion polymerase (Thermo Fisher, Waltham, Massachusetts, USA) according to manufacturer’s protocol with three different extension times (2.5, 5, and 10 min). The resulting amplicons were purified using MSB SPIN PCRAPACE (Invitek Molecular, Berlin, Berlin, Germany). The concentrations were measured using Qubit dsDNA HS Assay kit (Thermo Fisher, Waltham, Massachusetts, USA). The library was prepared using Nextera XT kit (see NetoVIR protocol) with 5-min tagmentation, sequenced, and analyzed as described above.

### Determination of the complete nudivirus genome

First, we identified a few relatively short contigs which could be attributed to nudiviruses, the longest contig of length 16,645 bp was obtained from one sample (Lisnice11). To enhanced detection of DNA, the sample was sequenced again without WTA2 preamplification which includes reverse transcription. One sample contained more than 50,000 reads attributable to *Nudiviridae* contigs (Lisnice24). All obtained reads from three samples were co-assembled with setting of SPAdes as described above. Then, we mapped individual samples on the scaffold, used vRhyme to get bins, extended them from the assembly graph with binSPReader, pre-release (37). The extended nudivirus bin was checked with Virsorter2 v2.2.4 (38) and DRAM v1.4.6 (39) to ensure that all contigs we have gained had a majority of *Nudiviridae* alignments. Then, we tried to get cleaner reads to resolve some regions. We mapped the reads of these three samples on the bin of four contigs undoubtedly belonging to *Nudiviridae* and extracted mapped reads (with a mate in case of one read from pair mapping). We repeated the mapping but on scaffold (>1 kbp) gained in co-assembly and extracted unmapped reads. The reads were from the three samples, mapped on nudivirus and unmapped on anything else (scaffold from co-assembly of three samples, greater than 1,000 bp). With these reads, we tried assembly, the best was obtained with SPAdes v3.15.5, --metaviral -k 21, 33, 55, and 77 settings which resulted in three contigs, 76,482, 24,683, and 22,709 bp (= 123,874 bp).

Therefore, we design primers similarly as for AmFLV (available at GitHub, see Data Availability for link), on the ends of contigs aiming outward. We performed PCR with different combinations of primers with Phusion polymerase and using different elongation times. The obtained fragments were purified and the library using Nextera XT (tagmentation based on lengths of amplicons: 2, 3, and 7 min) was prepared and sequenced on MiSeq with reads 2 × 250 bp.

### Comparison with published data sets

After finding possible new viruses in our *de novo* data, we analyzed sequences obtained by NGS from studies which used protocol for sample processing that allows detection of both RNA and DNA viruses (40). FastQ files obtained in the study by Deboutte et al. (41) were pulled from the Sequence Read Archive (SRA) archive using prefetch and fastq-dump available from NCBI, and all non-redundant scaffolds from all the samples were made available on GitHub. Additionally, we used data from our previous study (24). The non-redundant scaffolds from both studies were classified with Diamond blastx against NR with --sensitive and -c 1.

The viral set we created for further analyses consisted of novel viral genomes detected in the current study combined with viruses found in the previous studies (24, 41) as described above. The viral set contains all the vMAGs/genomes. Reads from the studies were mapped on them using bwa-mem2 v2.2.1 (32), and the coverage was extracted using CoverM v0.6.1 (https://github.com/wwood/CoverM).

### Phylogeny, visualization, annotations, and data processing

We gathered NS1 proteins of *Densovirinae* available in the database (NCBI, 06.16.2022), and the data set was filtered several times for partial and misnamed proteins. NS1-superfamily region was identified using Batch CD-Search (42) and extracted in Python. For AmFLV, the sequences of DNA polymerases were randomly selected from each group of large viruses (https://www.ncbi.nlm.nih.gov/labs/virus/vssi/#/). For the described AmNV, the core genes of 13 nudiviruses were extracted (DNA polymerase B, DNA helicase, integrase, p47, lef-4, lef-8, lef-9, vlf-1, p74, pif-1, pif-2, pif-3, pif-4/19 kda, 38k, vp39, vp91, ac81); for outgroup, we used *Baculovirus* with the same set of genes but without integrase (see https://github.com/kadlck/NAZV19 for accessions) as described in reference (26).

Alignment was done with mafft v7.520 --auto (43), then it was trimmed with trimAl v1.4.1 --gappyout (44). The best model was determined with ModelTest-NG v0.1.7 (45), and the tree was built using Phyml v3.3.20220408 (46) using the model best suited for our data set. Visualization was done using iTOL (47).

Annotations of densoviruses, Apis mellifera filamentous-like virus, and Apis mellifera nudivirus were performed using BLAST in NCBI (48) and DRAM-v v1.4.6 (39). Schemes of the genomes were generated using the Python library DNA Features Viewer v3.1.2 (49) and pyCirclize v0.5.0 (https://github.com/moshi4/pyCirclize), and other libraries were used to process data (Pandas, Numpy, Biopython). The terminal repeats were predicted by RNA-fold as specified in reference (50). The genomes of AmFLV and AmNV were polished using Pilon v1.24 (51).

## RESULTS

### Sequencing statistics

The sequencing statistics are listed in Table 1. We gained more than 10M of reads per sample. Also, a large number of reads mapped back to our curated vMAGs, showing efficiency of our protocol.

**TABLE 1:** The most important sequencing statistics of the samples^*a*^.

Sample	Reads	% of remaining reads	% of assembled	% of mapped to host	% of viral reads	N50	N50/dehosted	Longest contig	Longest contig/dehosted
Lisnice11	41,440,100	74.99	95.88	57.95	25.31	2,126	1,972	44,207	44,207
Lisnice24	30,596,366	62.40	94.37	66.67	25.38	2,214	2,011	28,235	28,235
Lisnice333	22,914,070	78.46	99.16	19.03	88.86	1,675	1,047	8,256	8,255
Brdy1	55,271,986	73.82	92.85	40.76	35.67	2,021	2,125	103,615	43,060
Brdy2	32,128,740	71.95	92.56	45.01	35.10	2,429	2,279	49,872	44,479
Brdy3	30,099,480	49.37	89.81	28.58	54.30	2,839	2,483	54,474	32,004
Libechov11	39,834,524	69.30	92.84	37.24	28.49	2,218	2,186	64,021	63,918
Libechov14	18,261,622	70.76	96.92	90.57	6.63	2,908	2,845	31,936	31,936
Libechov6	18,149,762	74.32	94.54	44.83	28.76	2,126	2,115	59,551	47,373
Prasily1	13,634,908	82.14	99.59	6.61	99.00	1,325	1,406	5,956	5,956
Prasily2	15,098,038	81.19	99.61	8.39	95.61	3,632	3,720	30,652	30,652
Prasily3	45,293,424	78.05	98.48	49.74	43.63	1,984	1,967	42,461	42,461
VURV1	26,401,994	76.93	98.18	32.37	60.10	1,965	1,942	47,953	47,953
VURV5	33,526,926	75.66	97.59	33.31	68.74	2,085	1,879	18,154	15,974
VURV4	44,055,956	84.95	99.57	12.82	94.37	2,349	2,279	72,955	72,955
VURV7	27,161,514	84.68	99.71	15.45	87.60	1,703	1,669	7,761	7,742
VURV_H	18,125,694	78.71	97.11	29.24	66.73	1,707	1,652	22,873	27,430
VURV_D	11,303,362	87.06	99.88	11.30	99.26	4,987	4,846	5,359	5,358

^*a*^ Number of remaining reads after trimming, number of those which mapped back at scaffold, to the host genome and to identified viral sequences. See full Table on GitHub (https://github.com/kadlck/NAZV19). Statistics were done on contigs >1,000 bp.

### Parvoviridae

**Fig 2:**
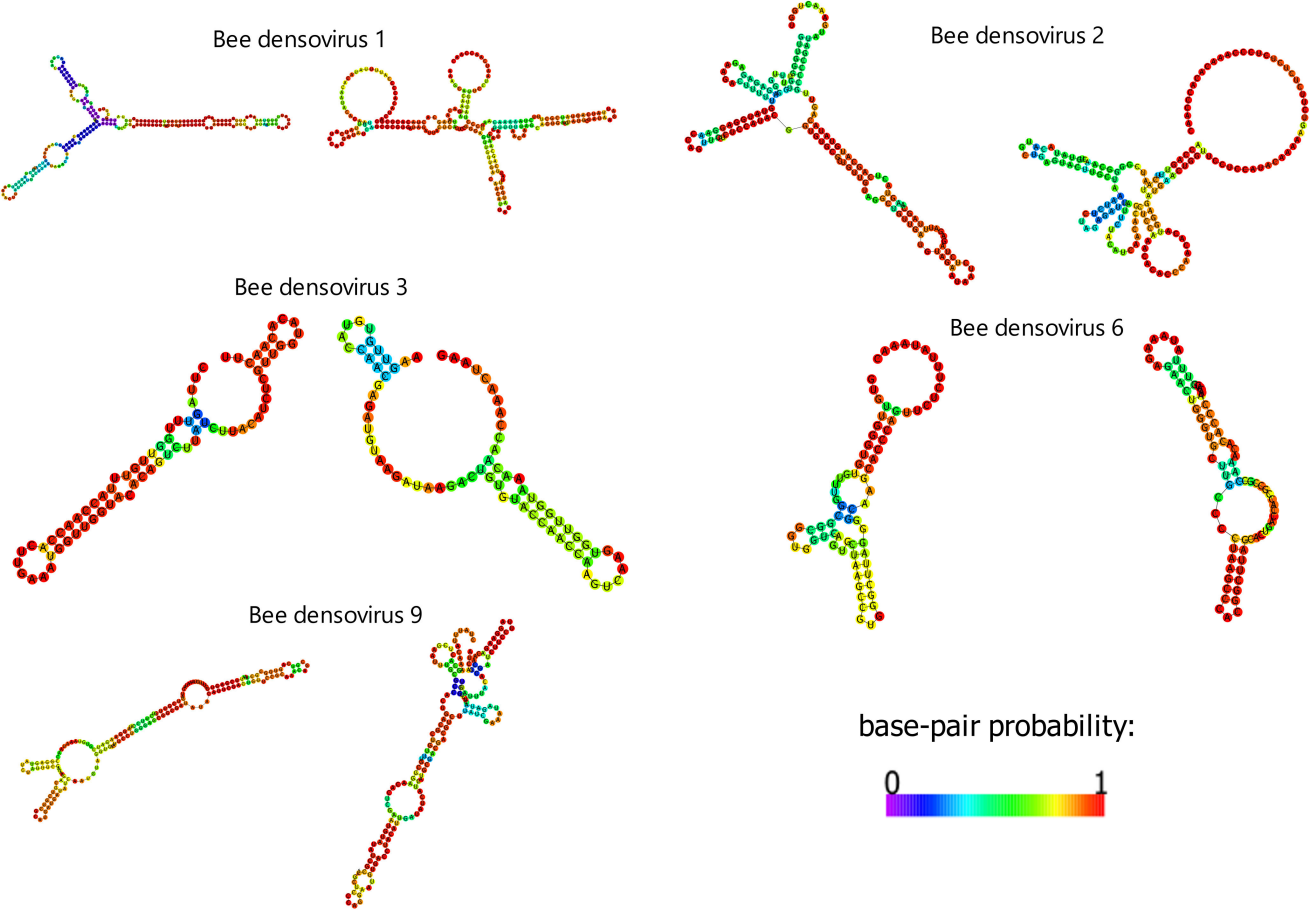
Predicted structures of terminal repeat from described densoviruses (RNA-fold program) as in reference (50). We can see familiar structures like Y-shape or I-shape. The color scale of pair-base probability is shown.

NGS of 18 analysed samples resulted in 77,423 reads which belong to the *Parvoviridae* family. This presents approximately 0.02% of all reads and 0.03% of those determined to be viral reads. These reads were present in 7 out of 18 samples. In the analyzed sequences from three studies from which we gained contigs, we found 16 unique contigs corresponding to the subfamily *Densovirinae*: nine were complete and seven were incomplete genomes. The complete genomes were named Bee densoviruses 1 to 9. The lengths of the complete genomes ranged from 3.6 to 6 kbp. All complete genomes contained two major ORFs that code structural and non-structural proteins with *Parvoviridae*-specific motifs and other short ORFs encoding additional hypothetical proteins surrounded by non-coding regions at both ends (Table 2; Fig. 2). The number of proteins and their predicted function based on similarity are shown in Table 3.

**TABLE 2:** Detailed information about the TRs and ends of the densoviruses^*a*^

Virus	TR length	TR type	TR mismatches (bp)	5´ Flank (before TR)	3´ Flank (after TR)
Bee densovirus 1	232	Homotelomeric	1	38	38
Bee densovirus 2	155	Homotelomeric	0	14	0
Bee densovirus 3	76	Homotelomeric	2	1	0
Bee densovirus 4	NP	NP	NP	14	104
Bee densovirus 5	NP	NP	NP	323	229
Bee densovirus 6	77	Homotelomeric	0	0	0
Bee densovirus 7	NP	NP	NP	414	223
Bee densovirus 8	NP	NP	NP	251	126
Bee densovirus 9	169	Homotelomeric	1	66	0

^*a*^ NP, not present.

**TABLE 3:** BLASTp results for complete densoviruses genome proteins^*a*^

ORF	Length (bp)	Length (aa)	Motif	Hit	Query coverage	E value	Percent identity	Accession
Bee densovirus 1 (4,865 bp) GC: 44.8%								
HP3	489	163	No hit					
SP	2,253	751		Structural protein (Tarsiger cyanurus ambidensovirus)	76%	2 × 10^−96^	38.6%	QTE04081.1
HP2	651	217	No hit					
NS1	1,554	518	NS1 motif	Non-structural ORFs (*Ambidensovirus* sp.)	88%	3 × 10^−68^	31.6%	AWV66973.1
HP1	723	241	No hit					
Bee densovirus 2 (5,601 bp) GC:39.4%								
NS1	1,635	545	NS1 motif	Non-structural protein 1 (Phylloscopus inornatus ambidensovirus)	99%	0	75.5%	QVW56790.1
SP-B	1,689	563	Denso_VP4	Structural protein VP1 (Phylloscopus inornatus ambidensovirus)	83%	0	70.8%	QVW56791.1
SP-A	960	320	Nterminal region of VP1 coat protein	Structural protein VP1 (Phylloscopus inornatus ambidensovirus)	81%	1 × 10^−53^	42.3%	QVW56792.1
NS2	819	273		Non-structural protein NS-2 (Culex pipiens densovirus)	100%	1 × 10^−97^	54.0%	YP_002887626.1
HP1	438	146	No hit					
HP2	414	138	No hit					
Bee densovirus 3 (3,632 bp) GC: 40.2%								
NS1	1,020	340	NS1 motif	TPA: MAG TPA: Rep 40 protein helicase (*Parvoviridae* sp.)	96%	3 × 10^−175^	69.1%	DAN51445.1
SP	1,611	537	Nterminal region of VP1 coat protein	TPA: MAG TPA: capsid protein (*Parvoviridae* sp.)	94%	4 × 10^−142^	45.2%	DAN51446.1
NS	486	162		TPA: MAG TPA: Rep 40 protein helicase (*Parvoviridae* sp.)	54%	4 × 10^−17^	51.1%	DAN51445.1
Bee densovirus 4 (5,160 bp) GC:41.8%								
SP	2,823	941	Nterminal region of VP1 coat protein	VP (uncultured densovirus)	60%	4 × 10^−33^	24.9%	QOD39535.1
NS1	1,533	511	NS1 motif	Putative non-structural protein (Phylloscopus schwarzi *parvoviridae* sp.)	82%	1 × 10^−40^	26.8%	QTE04075.1
HP2	1,023	341	No hit					
HP4	423	141	No hit					
HP3	498	166	No hit					
HP1	423	141	No hit					
Bee densovirus 5 (3,910 bp) GC: 40.2%								
SP	1,728	576		Structural protein (Phylloscopus ambidensovirus)	95%	6 × 10^−165^	48.7%	QTE03896.1
NS1	1,542	514	NS1 motif	Non-structural protein (Tarsiger cyanurus ambidensovirus)	85%	4 × 10^−83^	36.0%	QTE04079.1
HP	669	223	No hit					
Bee densovirus 6 (5,058 bp) GC: 42.0%								
SP-A	1,821	607	Denso_VP4	Structural protein VP2 (Tarsiger cyanurus *parvoviridae* sp.)	99%	0	93.7%	QVW56816.1
NS1	1,599	533	NS1 motif	Non-structural protein (bat-associated densovirus)	100%	0	91.9%	QOR29557.1
SP-B	741	247		Structural protein (Tarsiger cyanurus *parvoviridae* sp.)	100%	3 × 10^−164^	91.1%	QVW56817.1
HP2	417	139	No hit					
HP1	576	192	No hit					
NS2	789	263		Non-structural protein NS-2 (Blattella germanica densovirus 1)	95%	6 × 10^−117^	65.7%	NP_874382.1
NS	693	231		Non-structural protein (Nandayus nenday *parvoviridae* sp.)	100%	6 × 10^−155^	90.4%	QTE03739.1
Bee densovirus 7 (4,841 bp) GC: 38.3%								
NS2	855	285		Non-structural protein 2 (Grus japonensis *parvoviridae* sp.)	74%	2 × 10^−141^	94.3%	QTE03769.1
NS1	1,680	560	NS1 motif	Non-structural protein (bat-associated densovirus)	95%	0	57.5%	QOR29553.1
SP-B	765	255		Putative structural protein (Grus japonensis *parvoviridae* sp.)	96%	8 × 10^−45^	41.7%	QTZ83145.1
SP-A	1,722	574	Denso_VP4	VP1 (bat-associated densovirus)	99%	1 × 10^−172^	47.3%	QOR29554.1
Bee densovirus 8 (6,023 bp) GC: 41.5%								
HP1	708	263	No hit					
NS1	1,293	431	NS1 motif	Putative non-structural protein (*Ambidensovirus* sp.)	63%	6 × 10^−27^	29.6%	UGV24202.1
HP2	777	259	No hit					
HP3	543	181	No hit					
SP	2,862	954		Capsid protein (Emberiza spodocephala ambidensovirus)	24%	3 × 10^−6^	25.3%	QTE04116.1
Bee densovirus 9 (5,202 bp) GC: 40.4%								
HP1	597	199	No hit					
NS1	1,509	503	NS1 motif	Non-structural protein NS1 (*Densovirinae* sp.)	94%	5 × 10^−179^	52.1%	QJI53739.1
NS2	798	266		Non-structural protein NS-2 (Culex pipiens densovirus)	92%	9 × 10^−52^	40.0%	YP_002887626.1
HP2	513	171	No hit					
SP	2,553	851	Denso_VP4	Viral polypeptide VP1 (Diatraea saccharalis densovirus)	72%	6 × 10^−157^	45.1%	NP_046815.1

^*a*^ HP, hypothetical protein; NS, non-structural protein; SP, structural protein.

Terminal repeats (TRs) were present in five of the nine complete genomes (Bee densoviruses 1, 2, 3, 6, 9). Detailed information and predicted structure of TRs are shown in Table 2 ; Fig. 2 in the file with additional densoviruses figures at https://github.com/kadlck/NAZV19. The TRs were completely identical for Bee densoviruses 2 and 6 or had 1–2 bp mismatches for the other Bee densoviruses. The rest of the complete genomes lack terminal repetition; however, the ORFs are flanked by non-coding regions in length ranging from 14 bp (Bee densovirus 4) to 414 bp (Bee densovirus 7) at the 5´ end of the genomes and from 104 bp (Bee densovirus 4) to 229 bp (Bee densovirus 5) at the 3´ ends.

Incomplete genomes (3.7–4.5 kbp) contain neither non-coding regions nor complete ORFs. They were therefore deemed incomplete and are available on GitHub (see Materials and Methods for link).

**Fig 3:**
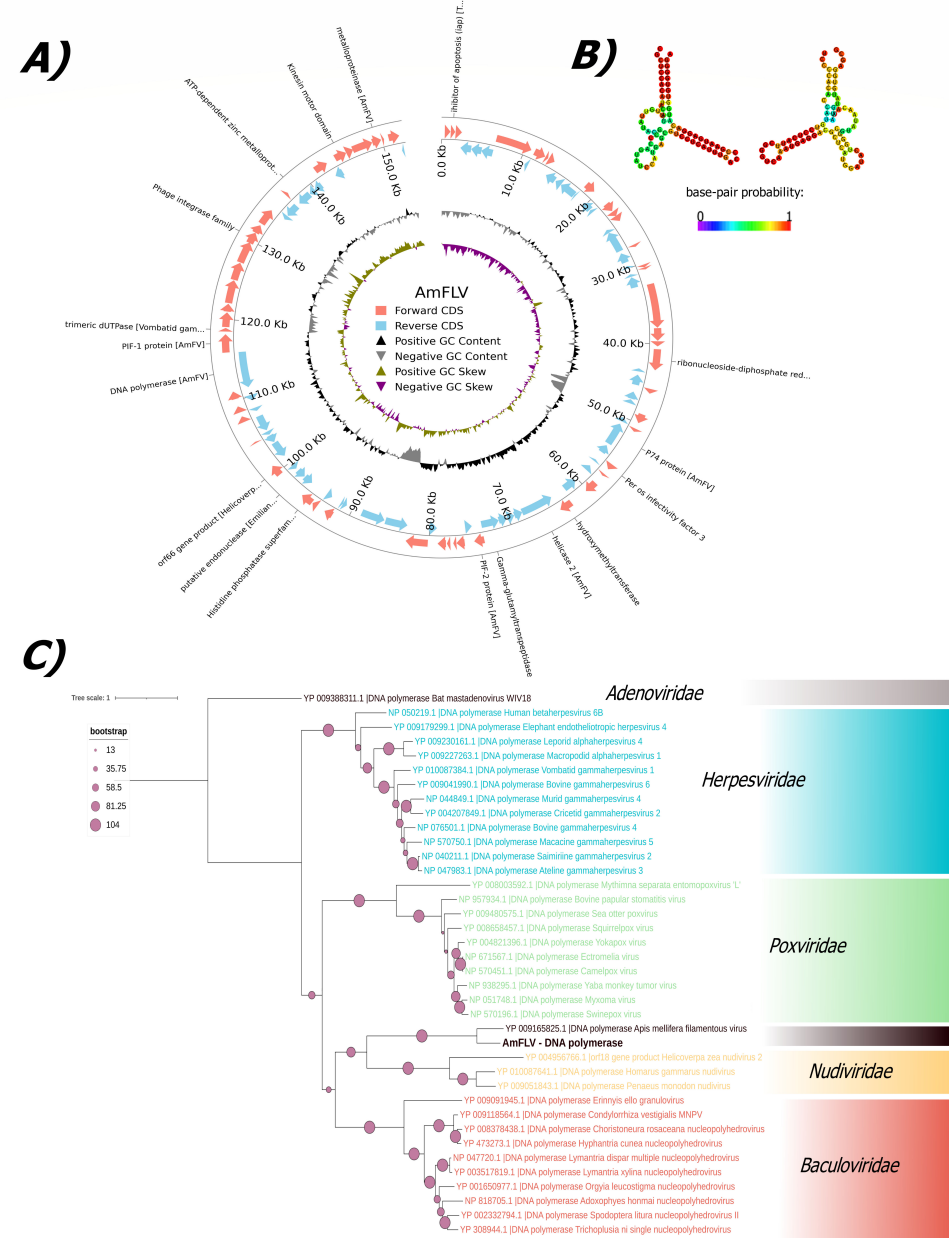
Information about the AmFLV. (A) Scheme of the genome. Only annotations that are not hypothetical proteins are shown with names. (B) Predicted structure of the TRs. The color scale of pair-base probability is shown. (C) Phylogenetic tree of AmFLV and random representatives of *Herpesviridae*, *Poxviridae*, *Nudiviridae*, and *Baculoviridae*.

The bee densoviral genomes were highly variable; they differed in length and terminal repetitions, and predicted ORFs. The sequence similarity of the genomes ranged from 24% to 44% across the whole genome (Table 2 in file with additional densoviruses figures at https://github.com/kadlck/NAZV19). This variability was confirmed by phylogenetic analysis using sequences retrieved from NCBI (Fig. 3 in file with additional densoviruses figures at https://github.com/kadlck/NAZV19). The genomes were distributed through the phylogenetic tree based on NS1-superfamily region-conserved sequences of densoviruses retrieved from NCBI. Bee densovirus 4 was closest to a member of *Miniambidensovirus,* Acheta domestica mini ambidensovirus (37.0% similarity over the whole sequence)*,* and Bee densovirus 7 to *Scindoambidensovirus* but also to members from the family *Densoviridae* (previously subfamily *Ambidensovirus*) that lack recent classification. Bee densovirus 8 was closest to sequences belonging to *Atrato Denso*-like viruses and Broome densovirus (31.2%, 30.3%, 31.2%). The group which included Bee densoviruses 1 and 5 had the highest similarity to *Ambidensovirus* sp., 40.6% for Bee densovirus 1 and 45.6% for Bee densovirus 5. Bee densovirus 3 was closest to Tarsiger cyanurus ambidensovirus with a similarity of 36.6% over the whole sequence. Bat-associated densovirus had a 50.6% similarity to Bee densovirus 7. Bee densovirus 6 had the highest similarity with 75.7% to Periparus ater ambidensovirus. For Bee densovirus 2, the similarity to Phylloscopus inornatus ambidensovirus was 59.9%. And finally, Bee densovirus 9 had a similarity of 46.7% to the *Ambidensovirus* sp.

### Apis mellifera filamentous-like virus (AmFLV)

From *de novo* sequencing, we identified one contig 103,615 bp long with low similarity to AmFV polymerase (30.8%). The retrieved sequence was extended as a vMAG with five more contigs (77,565 bp, 43,940 bp, 8,006 bp, 5,756 bp, and 4,443 bp), and CheckV predicted the vMAG as 92.9% complete. A ~10 kb amplicon was obtained using PCR with specific primers designed at the end of the large contigs (see GitHub, https://github.com/kadlck/NAZV19, for the scheme). After sequencing the amplicon, assembly, and polishing, a linear genome with TRs was obtained. Out of 39,950,966 reads, 448,034 reads (1.1% of the sample where contigs were discovered, region Brdy, specifically Brdy1) mapped to the complete genome. The complete genome length, provisionally named AmF-like virus, was 152,678 bp long with a GC content of 49.8%. A scheme of the genome is shown in Fig. 3A. The sequence of AmFLV was flanked by inverted homologous TRs 77 bp long, forming a Y-shape at both ends (Fig. 3B). A total of 112 ORFs were identified; the putative ORFs were distributed on both strands, and 49 (43.8%) were identified in protein databases (Viral, Peptidase, Pfam, Cazy, Vogdb, KEEG).

The predicted proteins mostly showed protein similarity to AmFV (similarity from 16.8% to 51.5%), but also other large viruses (see GitHub, https://github.com/kadlck/NAZV19, for all hits): protein numbered AmFLV_89 to trimeric dUTPase of Vombatid gammaherpesvirus 1 (55.1%), protein AmFLV_76 to orf66 gene product of Helicoverpa zea nudivirus 2 (58.4%), protein AmFLV_2 to inhibitor of apoptosis (iap) of Trichoplusia ni single nucleopolyhedrovirus (43.0%), AmFLV_34 to ribonucleotide reductase/HP APL35_gp114 of AmFV (50.4%), and protein AmFLV_74 to putative endonuclease of Emiliania huxleyi virus 86 (42.9%) and AmFLV_87 to DNA polymerase of AmFV (28.4%). Like other large viruses, this virus encodes its own DNA polymerase and proteins like dUTPase and metalloproteinase that can affect host cell metabolism. Additionally, this virus codes proteins like inhibitors of apoptosis or *per os* infectivity factors. On average, the identity of proteins with known function was higher than that of hypothetical proteins, and even higher for proteins that affect cell metabolism. However, even with several significant alignments (mostly to AmFV), most of the proteins are hypothetical or have no significant alignments detected.

Some characteristics of the new virus are similar to AmFV, like the presence of the *Baculoviridae*-related regions (pif-1/2/3), which are important for cell entry and are essential for *per os* infection. The similarities were relatively high (41.7%, 51.5%, and 41.7%) and we found pif 1–3 which form the conserved *per os* infectivity complex in *Baculoviridae* (52) or the presence of the kinesin motor domain which could be one of the components responsible for affecting cytoskeletal dynamics by viral infection. It may be important that the virus has significant similarity to one hypothetical protein of AmFV (APL35_gp042, 42.4%), which could encode integrase/recombinase closest to the phage integrase family (Pfam, Vogdb). The identified AmFLV virus was also detected in our previous study (24) and in the study by Deboutte et al. (41) (Table 4).

**TABLE 4:** Mapping of different samples from analyzed studies on three contigs belonging into one vMAG and to the whole AmFLV sequence completed by analyses of NGS available sequences and PCR*^a^*

Current study	Brdy1	Brdy2	Brdy3	Libechov11	Libechov6
AmFLV	448,034	213,635	20,568	2,113	1,851
NODE_1_length_103615_cov_192.460671	300,031	141,132	13,644	968	1,060
NODE_2_length_77565_cov_239.767590	552,407	252,903	23,319	2,608	2,195
NODE_4_length_43940_cov_193.303262	126,097	58,462	4,910	533	516

Kadlečková et al. (24)	BeeCelsaA2	BeeCelsaA3	BeeCelsaA4	BeeCelsaA5	BeeCelsaB3	BeeCelsaB4	BeeCelsaB5	BeeCelsaC3	BeeCelsaC4	BeeCelsaC5
AmFLV	1,606	7,663	715	2,214	45,358	636	960	5,355	859	37,731
NODE_1_length_103615_cov_192.460671	866	4,721	409	1,345	26,226	384	647	3,253	500	22,642
NODE_2_length_77565_cov_239.767590	792	5,252	542	1,362	22,194	277	1,082	4,082	454	23,236
NODE_4_length_43940_cov_193.303262	390	2,158	209	635	12,685	150	254	1,440	212	10,838

Deboutte et. al. (41)	SRR10418323	SRR10418338	SRR10418344	SRR10418344	SRR10418371	SRR10418385	SRR10418391
AmFLV	1,072	2,481	752	534	770	13,731	792
NODE_1_length_103615_cov_192.460671	286	1,649	488	325	175	8,797	566
NODE_2_length_77565_cov_239.767590	464	2,755	830	594	523	17,569	1,046
NODE_4_length_43940_cov_193.303262	192	726	226	134	175	3,861	188

^*a*^ The number of reads mapping is shown.

The phylogenetic analysis was done with a representative of large viruses (from NCBI, random selection from RefSeq DNA polymerases sequences in each family) and shows that the AmFV, based on sequences of polymerases, is the closest representative, but still clearly distinct (Fig. 3C).

The vMAG was made up mainly of three large contigs (103, 77, and 44 kbp), but only the 103 and 44 kbp contigs were mapped to the AmFLV. All three contigs have very similar mapping patterns (Table 4) across the samples where the contigs were present. The sequence of the third 77 kbp contig (made available on GitHub, https://github.com/kadlck/NAZV19) probably belongs to another large virus that infects honey bees. The inclusion of the 77 kbp contig in vMAG isn’t unexpected. The probability that the 77 kbp contig doesn’t contain a sequence of AmFLV is supported by the finding of a higher number of reads mapping to the shorter 77 kbp fragment in comparison to the 103 kbp contig and only two similarities to AmFV detected in the 77 kbp contig in comparison to dozens in the 103 and 44 kbp contigs (Table 4). Additionally, when we include the 77 kbp contig into vMAG, the two genes of ribonucleoside-diphosphate reductase were identified.

### Apis mellifera nudivirus (AmNV)

**Fig 4:**
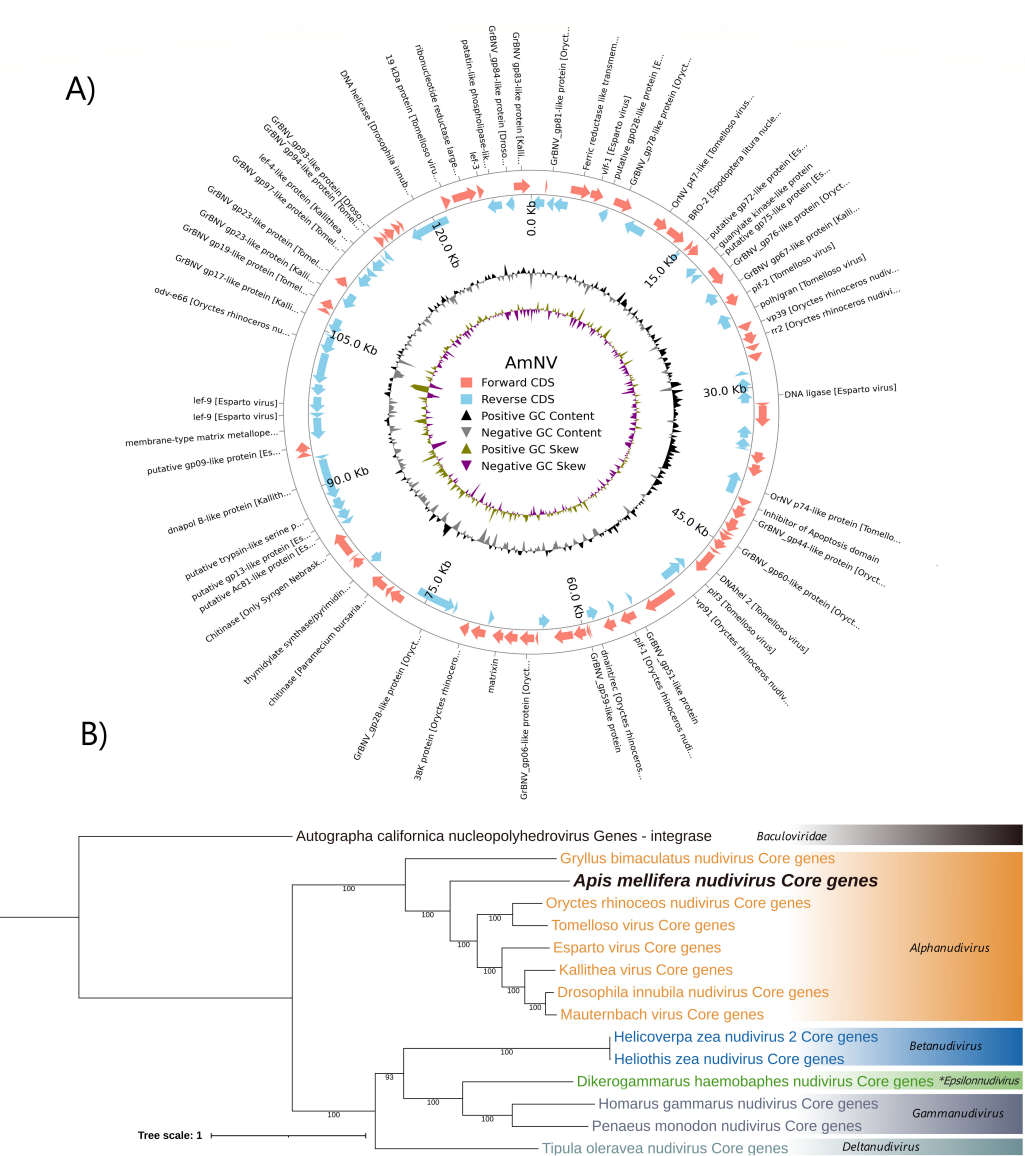
Information about new honey bee nudivirus. (A) Scheme of the genome. (B) Taxonomy of core genes * proposed genus (53).

At the beginning, we had 12 contigs which we reduced to three contigs by co-assembly (see Materials and Methods), 76,482, 24,683, and 22,709 bp (= 123,874 bp) with high reliability and continuity. They all had a number of hits to *Nudiviridae*. With primers that we designed at the ends of these contigs, and directed outward utilizing PCR, we were able to obtain three amplicons (<500 bp, <900 bp, <10 kbp) in reasonable combinations which make the virus circular and complete (see GitHub, https://github.com/kadlck/NAZV19, for the scheme). These amplicons were sequenced and the genome of the new nudivirus found in honey bees was completed. We gained a circular genome of 129,467 bp, coding 106 proteins on both strands of the viral genome. The virus had 40.3% GC content, was named Apis mellifera nudivirus, and taxonomically belongs to *Alphanudivirus* (Fig. 4). On the virus, mapped 219,147 reads out of 31,076,599 (0.7%) Lisnice 11 from sample sequenced using the full NetoVIR protocol (RNA/DNA preparation), 110,763 out of 13,409,876 (0.8%) Lisnice11 with only DNA sequenced (no WTA2 preamplification), and 67,247 out of 19,091,873 (0.4%) Lisnice24. The virus was also detected in other samples, but the number of mapping reads was below 50,000 reads and was also detected in studies which analyzed viromes of Czech (24) and Belgian bees (41).

Out of 106 proteins, 79 had some significant alignments (74.5%), mainly to four viruses: Oryctes rhinoceros nudivirus, Esparto virus, Kallithea virus, and Tomelloso virus which are all *Alphanudivirus*. The other significant alignments were AmNV_10 to BRO-2 of Spodoptera litura nucleopolyhedrovirus II (11.8%), AmNV_39 to inhibitor of apoptosis 3 of Choristoneura rosaceana entomopoxvirus “L” (27.8%), AmNV_64 to hypothetical protein APL35_gp193 of Apis mellifera filamentous virus (47.1%), AmNV_66 to chitinase of Paramecium bursaria Chlorella virus NYs1 (23.2%), another AmNV_70 to chitinase of Only-Syngen Nebraska virus 5 (26.6%), and AmNV_78 to membrane-type matrix metallopeptidase-1 of Anopheles minimus iridovirus (27.2%). All annotations are available on GitHub (see Materials and Methods for link), and a schematic of the genome is shown in Fig. 4.

The AmNV contains significant alignments to 17 core genes of the *Nudiviridae* family: AmNV_74 to DNA polymerase to Kallithea virus (42.9%), AmNV_49 integrase/recombinase of Oryctes rhinoceros nudivirus (51.9%), AmNV_41 to helicase 2 of Tomelloso virus (40.2%), AmNV_92 lef-4 (subunit DdRp) Kallithea virus (45.6%), AmFV_44 to lef-8 (catalytic subunit DdRp) of Kallithea virus (58.9%), AmNV_80 lef-9 (subunit DdRp) Esparto virus (51.9%), AmNV_46 to pif-1 Oryctes rhinoceros nudivirus (49.3%), AmNV_17 to pif-2 Tomelloso virus (68.3%), AmNV_42 to pif-3 of Tomelloso virus (58.2%), AmNV_5 to vlf-1 of Esparto virus (41.9%), AmNV_19 vp39 Oryctes rhinoceros nudivirus (53.7%), AmNV_33 to p74 of Tomelloso virus (50.6%), AmNV_43 vp91 of Oryctes rhinoceros nudivirus (36.1%), AmNV_61 to 38K Oryctes rhinoceros nudivirus (47.7%), AmNV_101 19 kDa protein Tomelloso virus (65.2%), and AmNV_71 to Ac81 protein of Esparto virus (49.5%). These core genes had, on average, greater similarity than the other significant alignments. We did not find any similarity for 4 out of 29 core genes (lef-5, p6.9, fen1, and 11K-like protein).

The virus contains a complete core of *per os* infectivity complex (pif-1/2/3) and all genes we expect to see in a member of *Nudiviridae* (core genes), mainly DNA polymerase B, integrase/recombinase, DNA-directed RNA polymerase, and DNA helicase 2. Apart from *Nudiviridae* proteins, AmNV has other significant alignments, which might be specific for honey bees (like alignment to APL35_gp193 of AmFV).

## DISCUSSION

Historically, honey bees have been thought to be primarily associated with a plethora of RNA viruses, belonging to the order *Picornavirales*, together with the most common members of *Iflaviridae* and *Dicistroviridae*. In stark contrast, only a handful of DNA viruses have been documented in this managed pollinator (18, 19). Our analysis in this study of 18 NGS samples, each representing a pool of 50 bees, together with sequences from two other studies (24, 41), has significantly expanded this DNA virome landscape that may be present in different honey bee populations. In our previous article, we suggested to use a number of pooled bees per sample, because this approach should allow to detect the diversity of honey bee viruses in the colony, and also low prevalence viruses (i.e., those present in only part of bees in a colony). Even if the viruses are in low abundance in the pooled sample, they can be used for *de novo* genome assembly (24). *de novo* assembly and the subsequent generation of vMAGs from the sequences performed in this study revealed several new DNA viruses. The benefit of analyzing larger numbers of bees can be documented by the detection of low prevalence viruses and proves to be beneficial in reflecting the diversity of DNA viruses in honey bees.

Most of the new DNA viruses could be classified as members of the *Densovirinae* subfamily. Densoviruses have a classical structure, two coding ORF cassettes and untranslated regions at the ends, with five out of nine having terminal repeats while others lack them. Not all known members of *Densovirinae* have terminal repetition at their genome ends (50). Our constructed phylogenetic tree was based on the conserved domain of NS1 even though the most described genera of *Densovirinae* clustered together, group previously classified as *Ambidensovirus,* and encompassing all the new -ambidensoviruses, were distributed through the tree with low bootstrap support. Members of the group are known to have even less than 30% similarity within the genus (22). The latest revision of the *Parvoviridae* taxonomy split the family into seven more lineages, some of which having greater similarity to *Iteradensovirus* than to other members of the group previously classified as *Ambidensovirus* (22).

The impact of *Densovirinae* on arthropods varies, ranging from overt pathologies (20, 54) to mutualistic relationships (55). Most members of the subfamily *Densovirinae* cause lethal infection of their hosts. The first symptoms are often anorexia and lethargy, followed by flaccidity, progressive paralysis, slow melanization, and tumor development (20). With a well-defined and distinguishable set of symptoms and a high viral load in a sick or dead animal, these viruses were relatively easy to identify before the era of NGS. Our bees that were selected for the analysis showed no overt signs of infection, such as deformities. To determine whether the newly described viruses are truly asymptomatic will require further study. The large number of found densoviruses with a low degree of nucleic acid similarities (23.9% to 44.4%) in two European countries (Czech Republic, Belgium) may indicate the presence of these and similar viruses also in other countries. The diversity of *Densovirinae* seems to be steadily increasing and new genera are being identified within the viruses previously known as ambidensoviruses. This high variability may not be limited to honey bee viruses like the *Densovirinae*. For instance, a recent study identified nine complete and six incomplete new species of chaphamaparvoviruses in six chickens (56).

After the initial analysis of the obtained sequences, we found a long fragment with low identities to AmFV. With viral binning, we found possible other assembly fragments of the new DNA virus. The virus AmFLV was completed using PCR that connected two contigs in one linear viral genome with TRs and the length over a 152 kbp. The terminal repeats form Y-structures at the ends of the genome. It contains a wide range of ORFs encoding a range of proteins from polymerase to those that affect the host cytoskeleton. Notably, the newly described AmFLV contains all three pifs *(1, 2, 3)* which have been documented as the core of the *per os* infectivity complex in baculoviruses (52). Apart from that, of interest might be the homology of the viral sequence with the phage integrase family and probable integrase/recombinase. This protein is also present in AmFV but denoted as hypothetical. It is possible, that under certain conditions, the virus is able to utilize this protein for integration into the host genome. The protein has been described in some large viruses, for example, in *Nudiviridae*. Therefore, it could be involved in establishing of latency and integration into the host genome (26). Overall, AmFLV encodes 112 ORFs, but the function of most of them remains undescribed (hypothetical), even for those that we were able to find similarity, mainly to AmFV.

The remaining long contig has a very similar mapping pattern across all samples where the contigs were present. The contig is approximately 77 kbp in size. However, we didn’t succeed in gaining its complete genome. It is also possible that this large fragment represents part of the genome of another large honey bee virus. Similar large DNA viruses with the same mapping patterns could easily be sorted as part of the vMAG of interest; therefore, the generation of vMAGs is extremely helpful in gaining genomic sequences but should be confirmed by a wet laboratory approach for some uses.

We completed the genome of nudivirus of honey bees using a combination of vMAGs generation, co-assembly, and amplicon sequencing. We gained a circular genome 129,467 bp long that contains the core genes of the *Nudiviridae*. In particular, lef-4, lef-5, lef-8, lef-9, and p47 are important for transcription, whereas pif-1-3, pif-4/19 kDa, and p74 are necessary for infection. There are several proteins important for viral morphogenesis, 38K, vp91, vlf-1, ac81, and vp39. The core genes also include proteins necessary for replication/recombination and repair like DNApol-B, helicase, and integrase. There are other important genes involved in nucleotide metabolism and some with unknown function (26). It is not surprising that the virus lacks 4 of the 29 suggested core genes (26) since, until present, only few nudiviruses have been described and the list of core genes is still changing. The virus encompasses a total of 108 ORFs, with significant alignments of predicted proteins to *Nudiviridae*, more precisely to *Alphanudivirus* (36.1% to 68.3%). The virus contains some proteins with reliable alignments to other viruses, like one alignment to AmFV, nucleopolyhedrovirus, or entomopoxvirus, but the similarities are low (11.8% to 47.1%). Phylogenetic analysis also revealed that the virus belongs into *Alphanudivirus*. The pathologies of *Nudiviridae* vary from virus to virus and between host’s life stages. In insects, they can cause lethargy, weakness, malformations, stunted growth, reduced longevity, or fertility. They can also cause changes in the viscosity and color of the hemolymph (opalescent), or they can concentrate in the abdomen and form a “waxy plug” (26). However, further studies are needed to find out if a specific pathology linked to AmNV exists. Generally, the infection with these viruses is less symptomatic in comparison to *Baculoviridae*, and they seem to be restricted to certain cell types (26).

With the exception of two colonies showing varroosis symptoms, all other colonies used as source colonies for sampling were considered healthy, i.e., they built up quickly, had no damaged capping and no signs of overt disease, or *Varroa* infestation were observed. However, all the bees we selected for the virome analysis were free of malformation and showed no signs of overt pathology. However, it is not possible to completely exclude the possibility that individual bees may have signs of pathology that were not obvious and visible when the individual bees were selected prior to virome analysis. Further studies with different experimental design and sampling are needed to clarify this issue.

Even though the binning of sequencing data is regularly performed for bacterial and eukaryotic species, the binning and generation of MAGs is still a novel method for viruses. Only in recent years, new software that is designed for binning of viral sequences has been released. The tools like Coconet (57), vRhyme (34), and vamb (58) perform better for creating viral MAGs since they provide a larger number of cleaner bins with fewer misclassified contigs in comparison to other programs that are better suited for creating bacterial and eukaryotic MAGs (34, 57, 58). This was confirmed by testing on a limited number of samples as we tried MetaWRAP (59) and all the viral binning software mentioned above. For our data set, vRhyme (34) performed best, but it would be beneficial to have a bin refinement tool such as MetaWRAP (59) with a combination of different binning software for viruses. The vMAGs generated were instrumental in piecing together a significant portion of a new large viral genomes, which otherwise might have been overlooked since its similarities to large viruses affect the statistics (e.g., in CheckV) and are deemed very incomplete. The generation of vMAGs then allows for keeping maximum information while still filtering out very incomplete and poor fragments. It allows us to “connect” the contigs and treat them as fragmented genomes.

In conclusion, our study underscores the richness of the honey bee DNA virome, which was previously overshadowed by their RNA counterparts. The plethora of densoviruses identified, coupled with the discovery of the AmFLV with its predominantly hypothetical proteins, and first nudivirus in honey bee (AmNV), paves the way for deeper investigations into the ecological and pathological implications of these viruses in bee populations.

## Data Availability

The raw data of the current study were deposited in SRA under BioProject id PRJNA1008242 and the described densoviruses under accessions nos. OR553295-OR553303, AmFLV under OR553294 and AmNV under OR596894. Other files (incomplete *Parvoviridae*; third contig of the predicted vMAG of AmFLV; primers used in the completion of AmFLV and nudivirus; annotation tables for AmFLV, third contig and nudivirus; selection of proteins for nudivirus taxonomy) are available on GitHub (https://github.com/kadlck/NAZV19) together with some of the code (jupyter-notebooks).
